# Potential of Essential Oils as Penetration Enhancers for Transdermal Administration of Ibuprofen to Treat Dysmenorrhoea

**DOI:** 10.3390/molecules201018219

**Published:** 2015-10-07

**Authors:** Jun Chen, Qiu-Dong Jiang, Ye-Ming Wu, Pei Liu, Jun-Hong Yao, Qing Lu, Hui Zhang, Jin-Ao Duan

**Affiliations:** 1 Jiangsu Collaborative Innovation Center of Chinese Medicinal Resources Industrialization, Nanjing University of Chinese Medicine, Nanjing 210023, China; E-Mails: chenjun75@163.com (J.C.); qiudong_J@126.com (Q.D.J.); njzyydxwym@sina.com (Y.M.W.); liupiper@gmail.com (P.L.); 2 Pharmaceutical Research Laboratory, School of Pharmacy, Nanjing University of Chinese Medicine, Nanjing 210023, China; E-Mails: yjh2014312@sina.com (J.H.Y.); lqnjutcm@163.com (Q.L.); zh20150908@sina.com (H.Z.)

**Keywords:** permeation enhancers, transdermal drug delivery, ibuprofen, essential oil, dysmenorrhoea, Chuanxiong oil

## Abstract

The present study was conducted to evaluate and compare five essential oils (EOs) as penetration enhancers (PEs) to improve the transdermal drug delivery (TDD) of ibuprofen to treat dysmenorrhoea. The EOs were prepared using the steam distillation method and their chemical compositions were identified by GC-MS. The corresponding cytotoxicities were evaluated in epidermal keartinocyte HaCaT cell lines by an MTT assay. Furthermore, the percutaneous permeation studies were carried out to compare the permeation enhancement effect of EOs. Then the therapeutic efficacy of ibuprofen with EOs was evaluated using dysmenorrheal model mice. The data supports a decreasing trend of skin cell viability in which Clove oil >Angelica oil > Chuanxiong oil > Cyperus oil > Cinnamon oil >> Azone. Chuanxiong oil and Angelica oil had been proved to possess a significant permeation enhancement for TDD of ibuprofen. More importantly, the pain inhibitory intensity of ibuprofen hydrogel was demonstrated to be greater with Chuanxiong oil when compared to ibuprofen without EOs (*p* < 0.05). The contents of calcium ion and nitric oxide (NO) were also significantly changed after the addition of Chuanxiong oil (*p* < 0.05). In summary, we suggest that Chuanxiong oil should be viewed as the best PE for TDD of ibuprofen to treat dysmenorrhea.

## 1. Introduction

The skin, the largest organ of the body, forms an effective barrier between the organism and the outer environment preventing pathogens invasion and fending off chemical and physical assaults [1]. It is also the most easily accessible organ of the body, making it a desirable site for both topical and systemic delivery of drugs. Consequently, transdermal drug delivery (TDD), the delivery of drugs across the skin, is gaining wide acceptance among pharmaceutical companies and medical professionals. TDD offers a number of advantages over conventional oral administration: (1) peak and valley levels in the blood are avoided; (2) first-pass metabolism is avoided and the skin metabolism is relatively low; (3) less frequent dosing regimens are needed due to the maintenance and longer sustainability of zero-order drug delivery and (4) less inter-subject variability and improving patient compliance are expected [2].

However, the stratum corneum (SC), the outermost layer of the skin, is the principal limitation to TDD. The SC is well recognized as the barrier that protects underlying tissue from infection, dehydration, chemicals and mechanical stress. The SC is composed of 15–20 layers of keratinized epidermal cells with no nuclei and cell organelles. The structure of the SC itself can be explained in terms of the so-called “brick and mortar” model in which horny keratinocytes (corneocytes) represent the bricks while the intercellular lipids and water-retaining natural moisturizing factors act as the mortar [3]. It is generally accepted that the permeability properties of SC barrier mainly results from the intercellular lipid matrix which consists of about 13 species of ceramides, free fatty acids, and cholesterol in an equimolar ratio [4]. The intercellular lipid domain is widely considered to be the main pathway for penetration of most drug molecules through the SC [5]. Until now, there were only a few molecules known with specific physicochemical properties that can cross the SC barrier sufficiently.

One of the most widely implemented approaches to improve TDD is the use of permeation enhancers (PEs), which ideally cause a temporary, reversible reduction in the barrier function of the SC in order to facilitate safe and effective drug delivery through the skin. Over the years, extensive screening and testing have identified different classes of chemicals as PEs, including Azone, sulphoxides, pyrrolidones, alcohols, surfactants, chelating agents as well as essential oils (EOs) and their constituents [6].

EOs, oily aromatic liquids extracted from aromatic plant materials, are natural products which consist of complex blends of many aromatic-smelling volatile compounds. The predominant compounds within these blends are terpenes, terpenoids, phenylpropanoids, as well as minor amounts of miscellaneous volatile organic compounds. EOs which possess diverse and relevant biological activities have been widely used for several applications in pharmaceutical, cosmetic, agricultural, and food industries [7].

As PE, EOs can increase the delivery of small drug compounds into the skin by interacting with the intercellular lipids through physical processes including extraction, fluidization, increased disorder, and phase separation. While EOs and their constituents can easily penetrate through the skin into the blood stream, they are also easily excreted from the body with urine and feces. Hence, natural EOs have increasingly been used due to their better safety profile compared to other PEs [6]. Furthermore, it was found that the enhancement permeation capacities of the whole EO were significantly higher than its main terpene components [8,9].

Dysmenorrhoea, which is caused by female endocrine disorders, is one of the unresolved problems in medical science. Approximately 80% of women globally had dysmenorrhoea in different degrees. Dysmenorrhoea is mainly characterized by cramping pain in the lower abdomen immediately before or during menstruation, which does affect the quality of their life and work.

Ibuprofen is a classical non-steroidal anti-inflammatory drug (NSAID) which is very effective for the symptomatic treatment of dysmenorrhoea [10,11]. Unfortunately, after oral administration, NSAIDs cause an increased risk of serious gastrointestinal adverse drug reactions including bleeding, ulceration, and perforation of the stomach or intestines, which can be fatal. Therefore, TDD of NSAIDs, such as ibuprofen, presents a safer potential alternative to oral therapy, which reduces the adverse side effects and avoids the hepatic first-pass metabolism. Recently, increasing attention has focused on TDD of ibuprofen [12]. Unfortunately, it is difficult to maintain effective blood concentrations of ibuprofen due to its poor skin permeability [13]. Several EOs have been applied to enhance TDD of NSAIDs [14,15,16]. It should be noted that EOs also have a broad biological activity spectrum. In the present study, the selected EOs possessing analgesic activity were Angelica oil, Chuanxiong oil, Cyperus oil, Cinnamon oil and Clove oil. The chief constituent of Angelica oil and Chuanxiong oil was ligustilide, which can attenuate pain [17]. Aromatherapy abdominal massage using EOs including Cinnamon oil and Clove oil was found to be effective in alleviating menstrual pain and excessive menstrual bleeding [18]. In addition, Cyperus oil also shows good analgesic effect [19]. The objective of the study was to evaluate and compare the five EOs as PEs to improve the TDD of ibuprofen. In our opinion, both the skin permeation enhancement and synergistic therapeutic efficacy will make the EOs become the potent PE for TDD of ibuprofen in the treatment of dysmenorrhoea. However, to the best of our knowledge, no studies have been performed to compare the skin permeation enhancement capacity of different EOs with the similar pharmacological activity.

## 2. Results and Discussion

### 2.1. Chemical Compositions of EOs

Extracted by the steam distillation method, the yield ratios (%) of Angelica oil, Chuanxiong oil, Cyperus oil, Cinnamon oil and Clove oil were determined to be 0.39% ± 0.08%, 0.61% ± 0.13%, 1.20% ± 0.06%, 3.57% ± 0.40% and 13.80% ± 0.31% (n = 3), respectively. The constituents of the five EOs (Angelica oil, Chuanxiong oil, Cyperus oil, Cinnamon oil and Clove oil) identified by GC-MS are shown in Table 1, Table 2, Table 3, Table 4 and Table 5, respectively. The major components of Angelica oil, Chuanxiong oil, Cinnamon oil and Clove oil were ligustilide (78.44%), ligustilide (41.28%), *trans*-cinnamaldehyde (82.75%) and eugenol (80.47%), respectively. For Cyperus oil, the main components were cyperene (27.69%), dehydrofukinone (27.54%) and α-cyperone (7.94%).

**Table 1:** Chemical composition of Angelica oil.

Peak No.	Retention Time (min)	Identified Compound	CAS	Relative Content (%)
1	6.252	(1*S*)-(−)-α-Pinene	7785-26-4	0.81
2	9.602	*trans*-β-Ocimene	3779-61-1	4.97
3	12.724	(4*E*,6*Z*)-2,6-Dimethyl-2,4,6-octatriene	7216-56-0	0.13
4	13.680	6-Butyl-1,4-cycloheptadiene	22735-58-6	0.94
5	17.629	6-Undecanone	927-49-1	0.16
6	18.985	2-Methoxy-4-vinylphenol	7786-61-0	0.39
7	20.168	2,4,5-Trimethylbenzaldehyde	5779-72-6	0.55
8	20.287	Eugenol	97-53-0	0.69
9	20.767	α-Cubebene	17699-14-8	0.16
10	21.821	2-Isopropyl-5-methyl-9-methylene[4.4.0]dec-1-ene	150320-52-8	0.38
11	22.599	3-Methylenecycloheptene	34564-56-2	0.50
12	23.020	Acoradiene	24048-44-0	0.27
13	23.555	Chamigrene	18431-82-8	0.20
14	24.111	1,5,5-Trimethyl-6-methylenecyclohexene	514-95-4	0.70
15	24.376	(+)-Cuparene	16982-00-6	0.13
16	24.473	β-Bisabolene	495-61-4	0.27
17	25.138	(+)-β-Himachalene	1461-03-6	0.17
18	26.553	Espatulenol	6750-60-3	1.12
19	29.049	2-Chloro-1-(2,4-dimethylphenyl)-2-methyl-1-Propanone	54965-53-6	1.28
20	29.762	Butylidenephthalide	551-08-6	4.13
21	29.983	*N*,*N*'-Diacetyl-1,4-phenylenediamine	140-50-1	0.24
22	31.377	*trans*-3*n*-Butylidenephthalide	1000365-98-3	0.76
23	31.582	2-Propyl-phenol	644-35-9	0.63
24	32.290	Ligustilide	4431-01-0	78.44
25	34.348	*trans*-Ligustilide	1000365-98-8	1.29

**Table 2:** Chemical composition of Chuanxiong oil.

Peak No.	Retention Time (min)	Identified Compound	CAS	Relative Content (%)
1	3.031	(+)-α-Pinene	7785-70-8	1.01
2	3.651	Sabenene	3387-41-5	0.69
3	4.518	α-Terpinen	99-86-5	0.87
4	4.684	*p*-Cymene	99-87-6	1.54
5	5.390	γ-Terpinene	99-85-4	2.69
6	5.999	Terpinolene	586-62-9	2.31
7	7.342	1(2*H*)-Naphthalenone,3,4,4a,5,8,8a-hexahydro-	14116-78-0	3.05
8	7.722	l-Terpinen-4-ol	20126-76-5	3.99
9	10.230	2-Methoxy-4-vinylphenol	7786-61-0	0.68
10	11.134	1-Phenyl-1-pentanone	1009-14-9	1.03
11	12.905	β-curcumene	1000374-17-4	2.04
12	14.702	β-Eudesmene	17066-67-0	6.16
13	14.974	α-Selinene	473-13-2	1.99
14	17.734	Espatulenol	6750-60-3	1.51
15	20.532	Methyl 4-ethylbenzoate	7364-20-7	4.95
16	21.334	Butylidenephthalide	551-08-6	7.12
17	21.580	*p*-Phenylenediacetamide	140-50-1	1.99
18	22.099	trans-Galbanolene	19883-29-5	1.28
19	23.324	Fenipentol	583-03-9	10.09
20	23.522	1,2,3,5,6,7-Hexahydro-inden-4-one	22118-01-0	3.74
21	24.003	Ligustilide	4431-01-0	41.28

**Table 3:** Chemical composition of Cyperus oil.

Peak No.	Retention Time (min)	Identified Compound	CAS	Relative Content (%)
1	4.479	trans-(−)-Pinocarveol	547-61-5	0.77
2	5.565	(−)-Myrtenol	19894-97-4	0.97
3	9.067	B-Damascone	35044-68-9	0.97
4	10.640	7-(1,1-Dimethylethyl)-3,4-dihydro-1(2*H*)-naphthalenone	22583-68-2	1.59
5	11.245	α-Cubebene	17699-14-8	1.08
6	12.337	Cyperene	2387-78-2	27.69
7	15.287	Rotundene	1000374-17-0	3.52
8	16.937	β-Eudesmene	17066-67-0	3.04
9	19.774	4-(4-Methoxyphenyl)iminopentan-2-one	20010-38-2	5.94
10	24.629	Caryophyllene oxide	1139-30-6	7.90
11	28.641	9H-Cycloisolongifolene, 8-oxo-	1000155-43-0	3.83
12	37.046	(−)-Isolongifolen-9-one	26839-52-1	3.95
13	38.874	Dehydrofukinone	19598-45-9	27.54
14	45.599	1,2,3,4,5,6-Hexahydro-1,1,5,5-tetramethyl-7*H*-2,4a-methanonaphthalen-7-one	23747-14-0	3.26
15	46.987	α-Cyperone	473-08-5	7.94

**Table 4:** Chemical composition of Cinnamon oil.

Peak No.	Retention Time (min)	Identified Compound	CAS	Relative Content (%)
1	4.239	Benzenepropanal	104-53-0	0.22
2	4.323	Borneol	507-70-0	0.10
3	5.157	3-Phenyl-2-propenal	104-55-2	0.47
4	6.249	trans-Cinnamaldehyde	14371-10-9	82.75
5	8.763	(+)-Cyclosativene	22469-52-9	0.24
6	9.078	α-Cubebene	17699-14-8	5.93
7	9.778	(+)-Sativene	3650-28-0	0.22
8	10.604	1-Caryophyllene	87-44-5	0.15
9	12.789	γ-Muurolene	30021-74-0	0.59
10	13.475	γ-Maaliene	20071-49-2	0.19
11	13.636	α-Muurolene	31983-22-9	2.16
12	14.000	1,2,4a,5,6,8a-Hexahydro-4,7-dimethyl-1-(1-methylethyl)naphthalene	483-75-0	0.17
13	14.224	d-Cadinene	483-76-1	3.61
14	14.441	1,6-Dimethyl-4-(1-methylethyl)-(1,2,3,4,4a,7)Hexahydronaphthalene	16728-99-7	1.37
15	14.714	1,1,6-Trimethyl-1,2-dihydronaphthalene	30364-38-6	0.25
10	17.746	t-Muurolol	19912-62-0	0.69
11	17.9	α-Copaene	3856-25-5	0.38

**Table 5:** Chemical composition of Clove oil.

Peak No.	Retention Time (min)	Identified Compound	CAS	Relative Content (%)
1	8.783	Eugenol	97-53-0	80.47
2	10.024	Caryophyllene	87-44-5	1.72
3	10.719	Humulene	6753-98-6	0.23
4	12.164	Eugenyl acetate	93-28-7	17.43
5	13.297	Caryophyllene oxide	1139-30-6	0.15

### 2.2. Cytotoxicity Assay of EOs on Skin Cells

**Figure 1:**
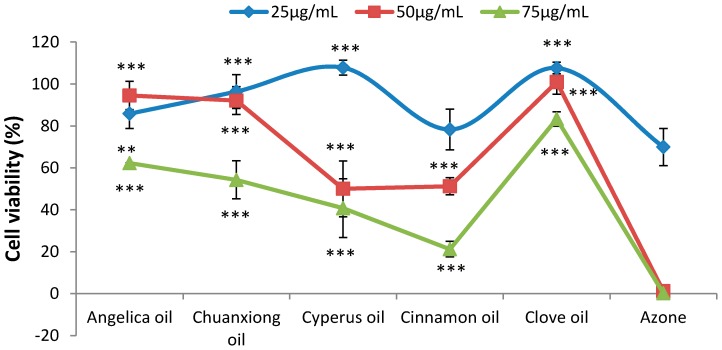
Cytotoxicities of EOs on HaCaT keratinocyte cell lines (n = 6). **p < 0.01, ***p < 0.001 *vs.* the Azone group.

The cytotoxicities of different analgesic EOs and Azone, the widely-used PE, were compared using an MTT assay. The results of keratinocytes treated with 25, 50 and 75 μg/mL EOs or Azone are shown in Figure 1. All examined PEs induced dose-dependent reductions in cellular viability. Compared with azone, the cytotoxicities of EOs were proved to be much lower. At the concentration of 50 μg/mL, the cell viability of Azone group was determined to be only 1.17 ± 0.23%. However, the viabilities of cells treated with EOs were all higher than 50%. The decreasing trend of cell viability at the concentration of 75 μg/mL was Clove oil >Angelica oil > Chuanxiong oil > Cyperus oil > Cinnamon oil >> Azone.

### 2.3. In vitro Skin Permeation

**Figure 2:**
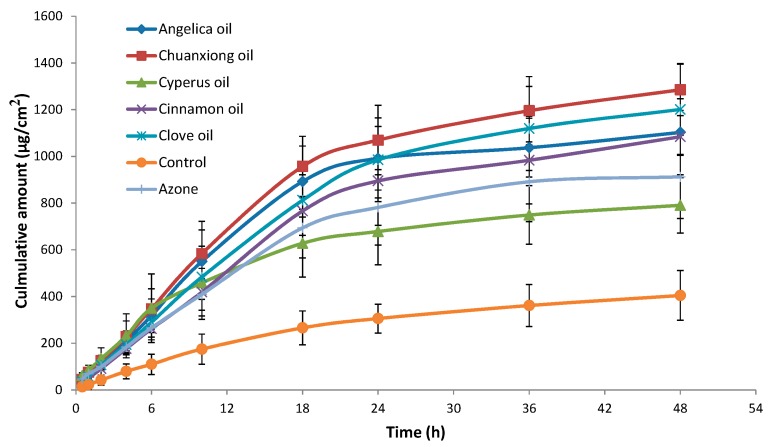
Skin permeation profiles of ibuprofen with various PEs through excised rat skin (n = 5). Control: ibuprofen hydrogel without PE.

Although the pig’s ear skin was considered to be the best model, due to the convenience of *in vitro* and *in vivo* comparison, rat skin was usually applied as skin model to investigate the skin permeation behavior [9,13,20]. The effect of EOs on the *in vitro* skin permeation profiles of ibuprofen through excised rat skin is shown in Figure 2. The permeation kinetics within 18 h followed zero order kinetics. The skin permeation parameters of ibuprofen with different EOs are listed in Table 6. All EOs significantly enhanced both the steady state flux and permeation ratio of ibuprofen compared to the control (*p* < 0.001). Among the EOs, the highest permeation rate of 52.05 ± 7.83 µg/cm^2^/h was obtained with Chuanxiong oil, while Cyperus oil produced the lowest enhancement in ibuprofen flux (32.97 ± 11.72 µg/cm^2^/h). Furthermore, except Cyperus oil, the ER values of EOs were all higher than that of Azone, especially Chuanxiong oil.

**Table 6:** Effect of PEs on the percutaneous permeation parameters of ibuprofen through excised rat skin (n = 5)

PE	Flux/µg·cm^-2^·h^-1^	ER	Q_48_/µg·cm^-2^	Permeation Ratio ^a^/%
Angelica oil	48.88 ± 8.92 ***	3.35	1102.57 ± 94.58 ***	57.70
Chuanxiong oil	52.05 ± 7.83 ***^,*#*^	3.57	1285.15 ± 109.92 ***^,*# #*^	67.26
Cyperus oil	32.97 ± 11.72 ***	2.26	790.28 ± 118.71 ***	41.35
Cinnamon oil	40.78 ± 9.99 ***	2.80	1084.21 ± 162.80 ***	56.74
Clove oil	44.70 ± 7.89 ***	3.07	1201.02 ± 197.02 ***^,*#*^	62.85
Azone	37.10 ± 8.51 ***	2.55	912.33 ± 178.13 ***	47.74
Control	14.57 ± 3.47	-	404.29 ± 106.10	21.16

^a^ Cumulative amount of permeated ibuprofen at 48h/added ibuprofen × 100%. Control: ibuprofen hydrogel without PE. ***p < 0.001 *vs.* the control group (ibuprofen only). *^#^* *p* < 0.05, *^# #^* *p* < 0.01 *vs.* the Azone group.

Among NSAID drugs, it was found that ibuprofen showed the poorest bioavailability. Following transdermal administration to rats, the area under the plasma–time curve (AUC) values of piroxicam, ketoprofen, naproxen, indomethacin and ibuprofen were determined to be 527.00, 269.45, 258.64, 243.22 and 88.08 μg/mL·h, respectively [13]. Therefore, it is indispensable to employ PE to increase the skin permeation of ibuprofen in order to maintain an effective blood level. Among the five EOs, only Clove oil had been evaluated as PE for TDD of ibuprofen hydrogel and the AUC values was increased by 2.4 times compared to the control [21]. In the present study, five analgesic EOs were compared as PE. Compared to the control (ibuprofen only), the ER values of ibuprofen *in vitro* were measured to be 3.35, 3.57, 2.26, 2.80, 3.07 and 2.55 using Angelica oil, Chuanxiong oil, Cyperus oil, Cinnamon oil, Clove oil and Azone as PE, respectively. As PE, EOs act mainly by disruption of the highly ordered structure of SC lipid with an increase in intercellular diffusivity [22]. And the intercellular spaces are the preferential pathway for lipophilic ibuprofen to penetrate through the SC.

### 2.4. Attenuated Total Reflection-Fourier Transform Infrared Spectroscopy (ATR-FTIR) Spectroscopic Studies

**Figure 3:**
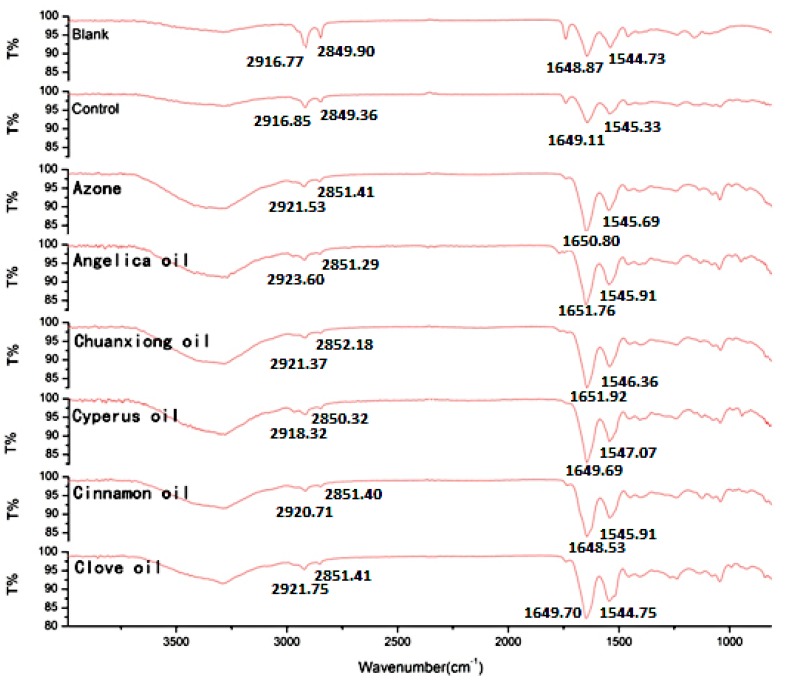
ATR-FTIR absorption spectra of rat skin treated with various PEs for 12 h. Note: the peak position values are the average of three replicates.

To elucidate the effect of EOs on the intercellular lipids in the SC, ATR-FTIR stretching peaks near 2850 cm^-1^ (C-H symmetric stretching absorbance frequency peak), 2920 cm^−1^ (C-H asymmetric stretching absorbance frequency peak), 1650 (Amide I) and 1540 (Amide II) were measured after the application of EOs to skin (Figure 3). The shift of a higher frequency occurs when the CH_2_ groups along the alkyl chain of lipids change from *trans* to *gauche* conformation, suggesting that the SC lipid is disturbed [23]. Peaks near 2920 cm^−1^ and 2850 cm^−1^, for asymmetric and symmetric stretching, respectively, showed that treatment of rat skin with the solvent (PG:IPA = 30:70 (*v*/*v*)) alone did not result in an obvious shift in stretching frequencies compared with untreated skin. In contrast, in skin treated with EOs or Azone, shifts were observed to CH_2_ symmetric and asymmetric stretching frequencies which were approximately 0.4 to 6.8 cm^−1^ higher than those observed with no treatment (blank) or with solvent alone (control). The peaks obtained near 2850 and 2920 cm^−1^ correspond to asymmetric and symmetric stretching modes, respectively, of the terminal methylene groups of the lipids, and these provided specific information about the interior composition of the lipid bilayer. The *trans*/*gauche* ratio of the alkyl tails affect the frequencies of the FTIR bands. A change in the band position from *trans* to *gauche* conformation indicates fluidization of the lipid bilayer. The magnitude of blue shift in the peak position of the asymmetric and symmetric stretching vibration absorbance is correlated with an increase number of *gauche* conformers in the lipid acyl chain. The higher the shifts, the higher the ratio of *gauche* to *trans* [24]. In summary, the results of ATR-FTIR studies revealed that EOs acted mainly by disruption of the highly ordered structure of SC lipid with an increase in intercellular diffusivity, which helped lipophilic ibuprofen to penetrate through the SC. In addition, according to the change in peak position of amide I or II, the five EOs also appeared to have a little effect on keratin, especially Chuanxiong oil and Angelica oil.

### 2.5. Pharmacological Experiments

**Figure 4:**
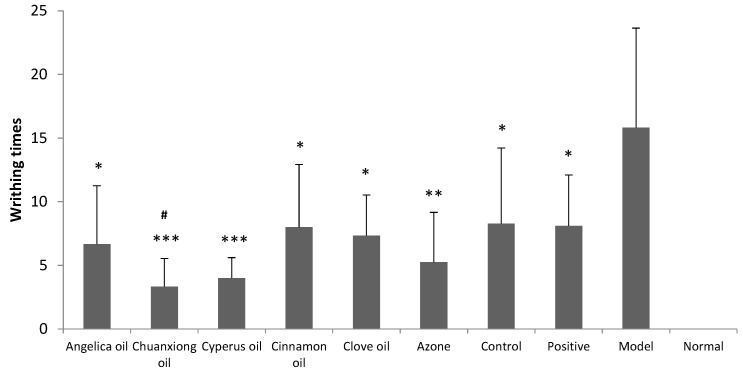
Inhibitory effects of ibu*p*rofen (140 mg/kg/d) with different EOs on dysmenorrheal model mice’s writhing times (n = 8). Positive: oral administration of 56 mg/kg/d ibuprofen suspension. Control: transdermal administration of ibuprofen hydrogel without PE. * *p* < 0.05, ** *p* < 0.01, *** *p* < 0.001 *vs.* the model group. *^#^ p* < 0.05 *vs.* the control group.

Results obtained from writhing test in dysmenorrheal model mice are shown in Figure 4. Model group and positive group were treated with blank hydrogel (transdermal administration) and the ibuprofen suspension (oral administration), respectively. Positive group, control group (transdermal administration of ibuprofen hydrogel without PE) and ibuprofen hydrogel containing different EO groups all produced significant decrease in the number of abdominal writhing response at the dose of 140 mg/kg/day (*p* *<* 0.05). The pain inhibitory intensity of ibuprofen hydrogel was obviously increased with Chuanxiong oil compared to the control (*p* < 0.05).

**Figure 5:**
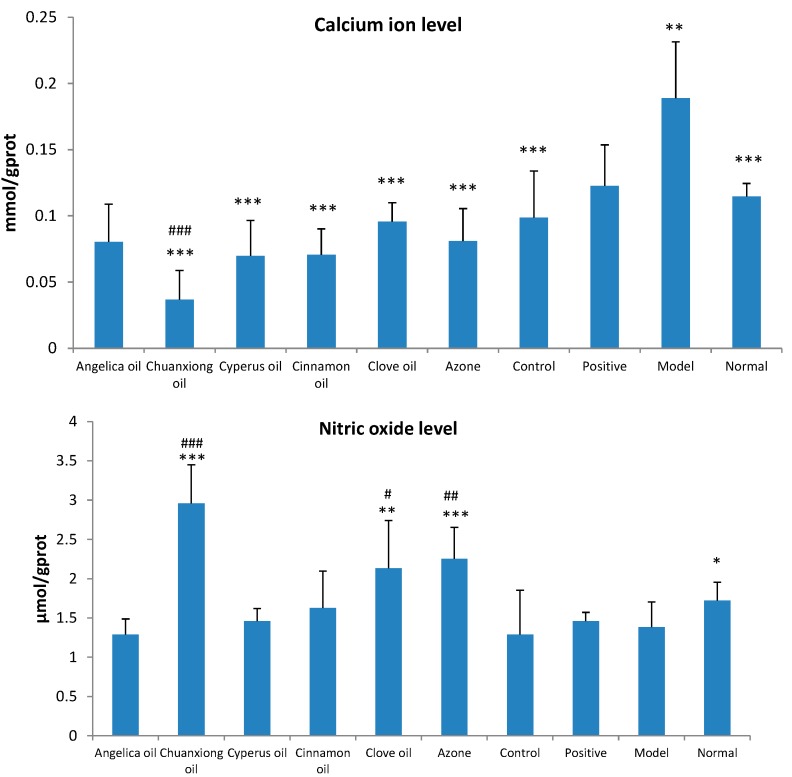

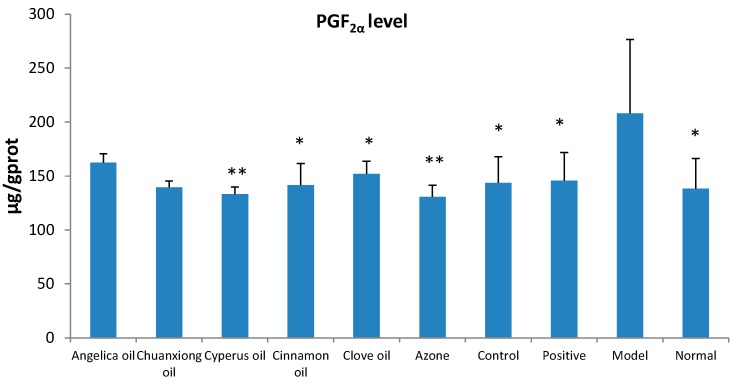
Effects of ibuprofen (140 mg/kg/day) with different EOs on the levels of calcium ion, nitric oxide and PGF_2α_ in uterine tissue of dysmenorrheal model mice (n = 8). Positive: oral administration of 56 mg/kg/day ibuprofen suspension. Control: transdermal administration of ibuprofen hydrogel without PE. * *p* < 0.05, ** *p* < 0.01, *** *p* < 0.001 *vs.* the model group. ^#^ *p* < 0.05, ^##^ *p* < 0.05, ^###^ *p* < 0.05 *vs.* the control group (ibuprofen only).

As shown in Figure 5, in primary dysmenorrheal model mice, the contents of Ca^2+^ and PGF_2α_ in uterus tissue homogenate were significantly increased (*p* < 0.05) and the level of NO was remarkably decreased (*p* < 0.05). The addition of EOs, especially Chuanxiong oil, could reduce the contents of Ca^2+^ and PGF_2α_ and increase the level of NO in uterus tissue homogenate.

Ibuprofen belongs to the NSAID family and exerts its main pharmacodynamic actions through inhibition of two isoforms of cyclooxygenase, COX-1 and COX-2. The analgesic, antipyretic, and anti-inflammatory activity of NSAIDs appears to operate mainly through inhibition of COX-2, whereas inhibition of COX-1 would be responsible for unwanted effects on the gastrointestinal tract [25]. COX enzymes are involved in inflammatory pathways and responsible for formation of pro-inflammatory prostaglandins. Prostaglandins which can cause constriction in uterine smooth muscle and sensitize spinal neurons to pain are regarded as the most pain factor to primary dysmenorrohea [26]. PGF_2α_, a naturally occurring prostaglandin, is used to induce labor in medicine. PGF_2α_ combined with its receptor on the spiral arterioles increased uterine contractility, resulting in ischemia pain [26]. In the present study, it was found that the PGF_2α_ levels in uterus were all significantly decreased after treatment with ibuprofen or ibuprofen with EO groups, especially for Chuanxiong oil groups which showed the highest enhancement effects in skin permeation studies of ibuprofen.

The levels of Ca^2+^ and NO in the dysmenorrheal model mice uterus were also detected. Overload of intracellular Ca^2+^ can cause uterine smooth muscle contraction and reduce the endometrial blood supply, leading to the occurrence of dysmenorrhoea. Therefore, calcium channel blocking agents decrease myometrial contractility and beneficial in cases of dysmenorrhea [27]. The treatment of ibuprofen, whether by oral or TDD route, all resulted in the significant decrease of Ca^2+^ levels, especially in combination with Chuanxiong oil. This indicated that one mechanism of ibuprofen treating dysmenorrhea may act on Ca^2+^ channel to decrease intracellular Ca^2+^ concentration. NO could regulate uterine contraction and execute pain modulation of peripheral and central levels [28]. As NO was negatively correlated with pain in acute inflammation [29], the significant increase of NO levels in uterus, which was observed in miceof Chuanxiong oil, Clove oil or Azone groups, contributed to inhibit analgesia. In summary, by comparing the writhing times and levels of PGF_2α_, Ca^2+^ and NO in uterus, TDD of ibuprofen in combination with Chuanxiong oil was found to be the most effective in the treatment of dysmenorrhea.

Rhizoma Chuanxiong, known as chuanxiong in China, is one of the most frequently used drugs in the prescriptions of traditional Chinese medicine for treating cardiovascular diseases, menstrual disorders and gynecological problems. Chuanxiong oil, rich in phthalide components, is generally claimed to play the major role in the heaemodynamic and analgesic effects of the herb. It had been demonstrated that Chuanxiong oil possessed potent analgesic and sedative activity [30,31] and was effective in relaxing vessels [32]. In addition, Chuanxiong oil had been proved to be a satisfactory PE for TDD of flurbiprofen [33], a lipophilic drug similar to ibuprofen.

It was noteworthy that a discrepancy between the percutaneous absorption and pharmacological studies, especially for Cyperus oil. The analgesic efficacy of Cyperus oil [19] seemed to be much higher than its permeation enhancement effect.

## 3. Experimental Section

### 3.1. Materials

Radix Angelicae Sinensis, Rhizoma Chuanxiong, Rhizoma Cyperi, Cinnamomum Cassia and Flos Caryophylli were all purchased from the Nanjing Medicinal Material Company (Nanjing, China). All the crude herbs were authenticated by the corresponding author. The voucher specimens (No. NJUCM 201407301~201407305) were kept in the Herbarium of Nanjing University of Chinese Medicine, Nanjing, China.

Ibuprofen and 3-(4,5-dimethylthiazol-2-yl)-2,5-diphenyltetrazolium bromide (MTT) were obtained from Sigma-Aldrich Inc. (St Louis, MO, USA). Azone was obtained from Sinopharm Chemical Reagent Co. Ltd (Shanghai, China). Carbopol 940 was obtained from Noveon Inc. (Cleveland, OH, USA). Acetonitrile was HPLC-grade from Merck (Darmstadt, Germany) and deionized water was purified by an EPED super purification system (EPED, Nanjing, China). Ibuprofen suspension was provided by Johnson Pharmaceutical Co. Ltd (Shanghai, China, No.141114087). The calcium ion (Ca^2+^), nitric oxide (NO) and prostaglandin F2α (PGF_2α_) test kits were obtained from Jiancheng Bioengineering Institute Co. Ltd. (Nanjing, China).

### 3.2. Extraction of the EOs

EOs were extracted by the steam distillation method. For each sample, the pulverized herb was accurately weighed and then transferred into a 1000-mL round-bottomed flask. Then a certain multiple (mL/g) of distilled water was added, and the sample was soaked for 1 h and subjected to 5–9 h of steam distillation using a Clevenger-type apparatus. Then the oil layer cooled to room temperature was transferred to a 5-mL measuring flask. A little anhydrous sodium sulfate was added to the sample to remove moisture. After centrifuge to remove sodium sulfate, the essential oil was stored in an air tight bottle and kept refrigerated until use. EOs from Radix Angelicae Sinensis, Rhizoma Chuanxiong, Rhizoma Cyperi, Cinnamomum Cassia and Flos Caryophylli were named Angelica oil, Chuanxiong oil, Cyperus oil, Cinnamon oil, and Clove oil, respectively.

### 3.3. GC-MS Analysis of the EOs

EOs were analyzed with GC-MS using an Agilent 7890A gas chromatograph interfaced to an Agilent 5975C inert MSD with Triple-Axis Detector. A NIST library was used for identifying the components. EO of 0.5 μL was injected into a HP-5 MS capillary column (30 m × 0.25 mm, 0.32 μm, i.d.) using a helium as gas carrier at 1 mL/min flow rate. Detailed parameters, such as inlet pressure, spilt ratio and temperatures of the column were described in Table 7. Mass spectra were recorded from 30 to 650 *m*/*z*. Individual components were identified by matching their 70 eV mass spectra with those of the spectrometer database as well as by comparison of the fragmentation pattern with those reported in the literature.

**Table 7:** Parameters of GC-MS analysis.

	Inlet Pressure/psi	Spilt Ratio	Temperatures of the Inlet/ °C	Temperatures of the Column
Angelica oil	7.6522	1:30	250	50 °C for 2 min, then 4 °C/min to 110 °C, then 5 °C/min to 140 °C, then 2 °C/min to 200 °C, then 8 °C/min to 250 °C for 10 min
Chuanxiong oil	10.523	1:30	200	80 °C for 4 min, then 10 °C/min to 125 °C, then 2 °C/min to 200 °C, then 50 °C/min to 250 °C for 5 min
Cyperus oil	10.523	1:30	150	100 °C for 3min, then 5 °C/min to 110 °C, then 1 °C/min to 120 °Cfor 20min, then 1 °C/min to 130 °C for 15 min, then 20 °C/min to 250 °C for 5 min
Cinnamon oil	7.6522	1:20	250	100 °C for 1 min, then 5 °C/min to 120 °C for 3min, then 2 °C/min to 130 °C, then 20 °C/min to 150 °C for 5min, then 20 °C/min to 200 °C for 5 min
Clove oil	10.523	1:20	200	100 °C for 3 min, then 6 °C/min to 200 °C, then 30 °C/min to 250 °C for 5 min

### 3.4. Cytotoxicity Assay

HaCaT (epidermal keratinocytes) cell lines were obtained from KeyGen Biotech Co. (Nanjing, China). The cells were incubated in minimum essential medium (MEM Eagles with Earle’s Balanced Salts) supplemented with 10% heat-inactivated fetal bovine serum (FBS) and 100 U/mL penicillin/streptomycin in a humidified incubator at 37 °C and 5% CO_2_.

The MTT assay was used to monitor the toxicity of EOs and Azone on human skin epidermal keratinocytes *in vitro* [9]. HaCaT cells were seeded into 96-well plates at a density of 7000 cells in a 100 μL medium per well. After 12 h, the cells were incubated with varying concentrations of EOs or Azone in a culture medium with 1% DMSO for 24 h at 37 °C. The cells that were treated with culture medium containing 1% DMSO were used as the control. Then the medium was replaced by a fresh medium containing 20 μL MTT solution (5 mg/mL in PBS) and the cells were incubated again for 4 h. Subsequently, the medium was removed and 150 μL DMSO was added to dissolve the formazan crystals. The plate was incubated for 10 min while shaking. The absorbance was measured at 490 nm using a Chromate-4300 microplate spectrophotometer (Awareness Technology Inc.; Palm City, FL, USA). Cell viability was calculated according to the following equation:

Cell viability = (A − B)/A × 100%
(1)

where A was the absorbance of the control and B was the absorbance of the cells incubated with different EOs or Azone, respectively. All samples were evaluated in sextuplicate.

### 3.5. Preparation of Ibuprofen Hydrogels

The gel was composed of ibuprofen (1% *w*/*v*), Carbopol (1%), ethanol (10%), EO or Azone (3%) and triethanolamine (1%). Carbopol powder was dispersed into the water phase and set for 24 h at room temperature, followed by adding triethanolamine for neutralization to form a homogeneous hydrogel. Ibuprofen mixed with EO or Azone was dissolved in ethanol. The alcoholic solution was slowly added to the vortex of agitated gel. The remaining water phase was added to the gel with continuous stirring. The contents of ibuprofen in hydrogels were measured by HPLC analysis (n = 3).

### 3.6. HPLC Analysis of Ibuprofen

A Shimadzu HPLC system (Kyoto, Japan) consisting of a LC-20AT pump, a SPD-20A UV-VIS detector was used for the assay of ibuprofen. The mobile phase consisted of acetonitrile and water (adjusted pH to 3.0 with phosphonic acid) (58:42, *v*/*v*). Separation was carried out at 25 °C using a reverse-phase C18 column (Inertsil ODS-3, 5 μm, 6 mm × 250 mm, Hanbang Corp.; Huaian, China). The detection wavelength was 220 nm and a flow rate of 1.0 mL/min was employed. A sample volume of 10 μL was injected.

### 3.7. Animals

Female ICR mice (18–22 g) and SD rats (180–220 g) were obtained from Shanghai Jiesijie Laboratory Animal Co. Ltd (Shanghai, China) with the license number SCXK (Shanghai) 2013-0006. They were housed in plexiglass cages at 22 ± 2 °C, relative humidity 55% ± 5% with 12 h light/12 h dark cycle and provided with standard pellet diet with tap water *ad libitum*. Animal experiments were performed in accordance to the Principles of Laboratory Animal Care and Use in Research (Ministry of Health, Beijing, China). The protocols of animal experiments were approved by the Animals Ethics Committee of Nanjing University of Chinese Medicine.

### 3.8. Skin Permeation Studies

After sacrificing the rats with excess diethyl ether inhalation, the abdomen skin fragment used for the experiment was excised from rats and the adhering fat and other tissues were removed. The full thickness skin prepared was subsequently washed with physiological saline solution three times and stored at −20 °C (used in two weeks). The skin was clamped between the donor and the receptor chamber of the Franz diffusion cell with an effective permeation area of 3.14 cm^2^ and a receiver cell volume of 8 mL. Physiological saline solution containing 40% ethanol was used as the receptor solution and incubated at 37 ± 0.2 °C using a water bath with a magnetic stirrer at 200 rpm. Different ibuprofen hydrogel formulations (0.6 g) were respectively gently spread onto the surface of skin in the donor chamber. Samples (0.8 mL) were withdrawn from the receptor chamber at predetermined time intervals (0.5, 1, 2, 4, 6, 10, 18, 24, 36 and 48 h) and then replaced with an equal volume of fresh medium. The receptor fluid samples were then analyzed by HPLC for ibuprofen content.

The cumulative amount of drug permeated through a unit area of skin was plotted against time. Steady state flux values were calculated from the slope of the linear portion of the plot (between 0 and 18 h). The cumulative amount of drugs permeating through the skin at 48 h (*Q*_48_) was calculated from the drug concentration in the receiver compartments. To compare the permeation enhancement capacities of each PE, the enhancement ratio (ER) was determined as follows: ER = (flux for skin treated with PE)/(flux for control).

### 3.9. ATR-FTIR Studies

After sacrificing the rats (n = 3) with excess diethyl ether inhalation, hair from the abdominal surface was removed with an animal hair clipper with an extreme precaution as to not impair the skin. The skin was then excised from the animals, the subcutaneous tissue was removed surgically and the dermal side was wiped with a cotton swab to remove the adhered fat tissue. The full thickness skin prepared was subsequently washed with normal saline, wrapped in aluminum foil, and stored at −20 °C (used within two weeks).

Attenuated total reflection-Fourier transform infrared spectroscopy (ATR-FTIR) spectrometry studies were performed in a similar manner to that reported previously [24]. The skin prepared as described above was cut into approximately 1 cm^2^ pieces and soaked in 10% *w*/*v* EO, 10% Azone or solvent (PG:IPA=30:70 (*v*/*v*), used as control) at 32 °C for 24 h, respectively. The treated skin samples were washed with distilled water and blotted dry. The infrared spectra of skin samples were obtained using Fourier transform infrared spectroscopy (FTIR-230 spectrometer, JASCO Co., Tokyo, Japan) with an ATR unit (ATR-500/M, JASCO Co.). The spectrum recorded represents an average of 32 scans obtained with a resolution of 2 cm^−1^ at room temperature. The spectra were collected in the wave number range of 4000~650 cm^−1^. The internal reflectance element (IRE) used in this study was a zinc selenide trapezoid having 45° entrance and exit faces. Skin was carefully mounted on the IRE.

### 3.10. Primary Dysmenorrhoea Mice Model and Administration

According to the previous reported method [34], estradiol benzoate and oxytocin were used to make dysmenorrheal mice model. Estradiol benzoate 0.01 g/kg/day was administrated to mice by subcutaneous injection for 12 days. On the thirteenth day, oxytocin 20 U/kg was administered by peritoneal injection.

ICR mice were divided into eleven groups. Normal control group was treated only with blank hydrogel. Model control group was treated with estradiol benzoate, oxytocin and blank hydrogel. For ibuprofen suspension (as positive control, 56 mg/kg/day, oral administration) and ibuprofen hydrogel groups (ibuprofen, ibuprofen + Angelica oil, ibuprofen + Chuanxiong oil, ibuprofen + Cyperus oil, ibuprofen + Cinnamon oil, ibuprofen + Clove oil or ibuprofen + Azone) at the dose of 140 mg/kg/day, the test medicine was administered for five days from the seventh day in modeling period. The number of writhing and stretching response was cumulatively counted from 0 to 30 min after the injection of oxytocin. The mice were then scarified. The content of PGF_2α_, Ca^2+^ and NO in the homogenate of uterus were determined according to specification of kits, respectively.

### 3.11. Statistical Analysis

The results were expressed as mean ± S.D. Statistical comparisons were made using Student’s *t*-test and the chosen level of significance was *p* *<* 0.05*.*

## 4. Conclusions

In the present paper, five analgesic EOs were compared with Azone to act as PE for TDD of ibuprofen. The results of skin cell cytotoxicity revealed that the safety of EOs were far higher than that of Azone. Moreover, the results of both skin permeation and pharmacological studies had demonstrated that Chuanxiong oil should be viewed as a potential PE for TDD of ibuprofen to treat dysmenorrhea. The pain inhibitory intensity of ibuprofen hydrogel was obviously increased with Chuanxiong oil compared to ibuprofen without EOs (*p* < 0.05). The contents of calcium ion and NO were also significantly changed after the addition of Chuanxiong oil (*p* < 0.05). In addition, the results of ATR-FTIR studies also demonstrated that EOs could disturb the highly ordered intercellular lipid structure between corneocytes in the SC.
